# European Stroke Organisation expedited recommendation for the use of short-term dual antiplatelet therapy early after minor stroke and high-risk TIA

**DOI:** 10.1177/23969873211000877

**Published:** 2021-03-11

**Authors:** Jesse Dawson, Áine Merwick, Alastair Webb, Martin Dennis, Julia Ferrari, Ana Catarina Fonseca, for the European Stroke Organisation

**Affiliations:** 1 College of Medical Veterinary and Life Sciences, University of Glasgow, Glasgow, UK; 2 Department of Neurology, Cork University Hospital, Cork, Ireland; 3 Wolfson Centre for Prevention of Stroke and Dementia, Department of Clinical Neurosciences, John Radcliffe Hospital, University of Oxford, Oxford, UK; 4 Centre for Clinical Brain Sciences, University of Edinburgh, Edinburgh, UK; 5 Department of Neurology, St. John's of God Hospital, Vienna Austria; 6 Department of Neurosciences (Neurology), Hospital de Santa Maria, University of Lisbon, Lisboa, Portugal

**Keywords:** Stroke, TIA, antiplatelets

## Abstract

Prevention of early recurrent ischaemic stroke remains a priority in people with TIA or ischaemic stroke. A number of trials have recently been completed assessing the efficacy of short-term dual antiplatelet therapy (DAPT) versus single antiplatelet therapy early after minor or moderate stroke or high-risk TIA. We present an Expedited Recommendation for use of dual antiplatelet therapy early after ischaemic stroke and TIA on behalf of the ESO Guideline Board. We make a strong recommendation based on high quality of evidence for use of 21-days of dual antiplatelet therapy with aspirin and clopidogrel in people with a non-cardioembolic minor ischaemic stroke or high-risk TIA in the past 24 hours. We make a weak recommendation based on moderate quality evidence for 30-days of dual antiplatelet therapy with aspirin and ticagrelor in people with non-cardioembolic mild to moderate ischaemic stroke or high-risk TIA in the past 24 hours.

## Introduction

Prevention of early recurrent ischaemic stroke remains a priority in people with TIA or ischaemic stroke. A number of trials have recently been completed assessing the efficacy of short-term dual antiplatelet therapy (DAPT) versus single antiplatelet therapy early after minor or moderate stroke and high-risk TIA.

The role of dual antiplatelet treatment will be considered in both the European Stroke Organisation’s guidelines for management of TIA and guidelines for secondary prevention of ischaemic stroke, yet their use is rapidly increasing. Therefore, in advance of their publication, we present an Expedited Recommendation for use of dual antiplatelet therapy early after minor ischaemic stroke and TIA on behalf of the ESO Guideline Board.

We considered evidence for use of DAPT early after minor or moderate ischaemic stroke and high-risk TIA. We considered DAPT with clopidogrel and aspirin separately from DAPT with ticagrelor and aspirin.

## Methods

This Expedited Recommendation was developed by a sub-group of the TIA Management and Stroke Secondary Prevention guidelines Module Working Groups (MWG). The composition of these groups was approved by the ESO Guidelines Board and the ESO Executive Committee, based on a review of the intellectual and financial disclosures of the proposed members. ESO guidelines follow GRADE methodology^1^ and the ESO Standard Operating Procedures.^2^ In brief, PICO (Population, Intervention, Comparator, Outcome) questions and further outcomes of clinical interest are identified. The relevance of possible outcomes is voted on by all members of the MWG using Delphi methods.

For each PICO question, systematic searches of PubMed, covering the period from the inception of each database to 15^th^ September 2020, were conducted by the ESO Guidelines methodologist, Avtar Lal (AL). Two authors independently screened the titles and abstracts of publications identified from the searches and assessed the full text of potentially relevant studies. Within one week of ictus was defined as ‘early’. This recommendation only considered data on adults.

The risk of selection, performance, detection, attrition and reporting biases in each randomised trial was assessed using the Cochrane Collaboration’s tool, and heterogeneity across studies was assessed using Cochran’s Q (reported as a p value) and I^2^ statistics. For each PICO question and each outcome, the quality of evidence was rated using the GRADEpro Guideline Development Tool (McMaster University, 2015; developed by Evidence Prime, Inc.) as high, moderate, low or very low. An evidence-based recommendation according to the GRADE evidence profiles and the ESO standard operating procedure was then developed and reviewed by all MWG members and modified using a Delphi approach until consensus was reached. The document was subsequently reviewed and approved by two external reviewers, and members of the ESO Guidelines Board and Executive Committee.

We developed 2 PICO questions;

1. In people with a non-cardioembolic minor ischaemic stroke or high-risk TIA, does early initiation of dual antiplatelet therapy with aspirin and clopidogrel, compared to aspirin monotherapy, reduce the risk of stroke recurrence?
2. In people with a non-cardioembolic mild to moderate ischaemic stroke or high-risk TIA, does early initiation of dual antiplatelet therapy with aspirin and ticagrelor, compared to aspirin monotherapy, reduce the risk of stroke recurrence?

## Summary of current evidence

Our literature search identified four RCTs^3–6^ which tested DAPT early after minor or moderate ischaemic stroke and TIA. Three RCTs (CHANCE, FASTER, POINT) tested the combination of aspirin and clopidogrel versus aspirin and placebo and have already been summarised in a systematic review and meta-analysis.^7^ Only people with minor ischaemic stroke (NIHSS ≤3) and high-risk TIA were included in these trials. Minor ischaemic stroke was defined as NIHSS of 3 or less in the POINT and CHANCE trials and high-risk TIA as an ABCD2 score of 4 or more. In the FASTER trial, people with TIA needed to have either weakness or speech disturbance as part of the symptom complex with a duration of 5 minutes or more to qualify for inclusion in the study.

One RCT (THALES) tested aspirin and ticagrelor versus aspirin and placebo in people with minor or moderate stroke and high-risk TIA.^6^

Patients with lower-risk TIA or only possible TIA or patients not assessed by a stroke specialist were not included in any of the identified randomised trials. In forming this recommendation we included outcomes up to 90-days after randomisation.

## Summary of data for PICO 1

- In people with a non-cardioembolic minor stroke or high-risk TIA, does early initiation of dual antiplatelet therapy with aspirin and clopidogrel, compared to aspirin monotherapy, reduce the risk of recurrent ischaemic stroke?

The three RCTs included a total of 10,447 patients. Compared with aspirin alone, dual antiplatelet therapy with clopidogrel and aspirin;

- significantly reduced the risk of ischaemic stroke at 90 days (RR 0.70, 95% CI 0.61 to 0.81, I^2^ = 0%, absolute reduction 2.6%, high quality evidence, n = 2 trials, 10051 participants).
- significantly reduced the risk of any stroke at 90 days (RR 0.71, 95% CI 0.62 to 0.82, I^2^ = 0%, absolute reduction 2.6%, high quality evidence, n = 3 trials 10244 participants).
- non-significantly increased risk of haemorrhagic stroke at 90 days (RR 1.18, 95% CI 0.53 to 2.65, absolute increase 0.1%, I^2^ = 0%, low quality evidence, n = 2 trials, 10051 participants).
- Non-significantly increased risk of major bleeding at 90 days (RR 1.73, 95% CI 0.69 to 4.30, I^2^ = 37%, absolute increase 0.2% moderate quality evidence, n = 3 trials, 10443 participants).

Ischaemic stroke was the most common stroke event and was more common than haemorrhagic stroke (total of 786 ischaemic strokes, 23 haemorrhagic strokes).

A pooled analysis of data from the CHANCE and POINT trials found that the benefit of dual antiplatelet therapy with clopidogrel and aspirin was confined to the first 21 days after minor ischaemic stroke and TIA.^8^ Moreover, during the chronic phase, large RCTs of aspirin and clopidogrel vs antiplatelet monotherapy including the CHARISMA,^9^ SPS2^10^ and MATCH^11^ trials, have not shown any net benefit; this is because any reduction in ischaemic events has been largely offset by bleeding, the risk of which is cumulative over time.

**Table 1:** 

Recommendation 1
In people with a non-cardioembolic minor ischaemic stroke (NIHSS score of 3 or less) or high-risk TIA (ABCD2 score of 4 or more) in the past 24 hours, we recommend 21-days of dual antiplatelet therapy with aspirin and clopidogrel, followed by antiplatelet monotherapy thereafter.
Quality of evidence: High ⊕⊕⊕⊕
Strength of recommendation: Strong for intervention ↑↑

## Summary of data for PICO 2

- In people with a non-cardioembolic mild to moderate ischaemic stroke or high-risk TIA, does early initiation of dual antiplatelet therapy with aspirin and ticagrelor, compared to aspirin monotherapy, reduce the risk of stroke recurrence?

The THALES study was the only identified trial and included 11,016 people with high-risk TIA or minor ischaemic stroke. Participants were given either ticagrelor plus aspirin or matching placebo plus aspirin. Outcomes were assessed at 30-days. Compared with aspirin alone, dual antiplatelet therapy with ticagrelor and aspirin;

- significantly reduced the risk of ischaemic stroke at 30-days (RR 0.79, 95% CI 0.68 to 0.93, absolute reduction 1.3%, moderate quality evidence).
- significantly reduced the risk of any stroke at 30-days (RR 0.81, 95% CI 0.69 to 0.95, absolute reduction 1.2%, moderate quality evidence).
- significantly increased risk of intracranial haemorrhage (HR 3.33, 95% CI 1.34 to 8.28, absolute risk increase 0.3%, low quality evidence).
- significantly increased the risk of severe bleeding (HR 3.99, 95% CI 1.74 to 9.14, P  =  0.001, absolute risk increase of 0.4%, low quality evidence).

**Table 2:** 

Recommendation 2
In people with non-cardioembolic mild to moderate ischaemic stroke (NIHSS of 5 or less) or high-risk TIA (ABCD2 score of 6 or more or other high-risk features*) in the past 24 hours, we suggest 30-days of dual antiplatelet therapy with aspirin and ticagrelor followed by antiplatelet monotherapy thereafter.
*defined as either intracranial atherosclerotic disease or at least 50% stenosis in an internal carotid artery that could account for the presentation.
Quality of evidence: Moderate ⊕⊕⊕
Strength of recommendation: Weak for intervention ↑?

**Table 3:** 

Expert consensus statement
In people with acute non-cardioembolic low risk TIA or in whom the diagnosis is uncertain, 11/11 experts voted against use of dual antiplatelet therapy over monotherapy

### Additional information

Across 3 trials, treatment with an aspirin and clopidogrel based DAPT regimen for 21 days after a high-risk TIA or minor stroke would be expected to prevent 26 ischaemic strokes for every 1000 patients treated but may not increase risk of intracranial haemorrhage.

In the one included study, treatment with an aspirin-ticagrelor based DAPT regimen for 30 days after high-risk TIA or minor or moderate stroke would be expected to prevent 13 ischaemic strokes for every 1000 patients treated but may be associated with 2 extra cases of haemorrhagic stroke.

## Considerations before starting DAPT

Brain imaging should always be performed prior to initiation of dual antiplatelet therapy.

Dual antiplatelet therapy should be started as soon as possible, within the first twenty-four hours after symptom onset.

The definition of minor ischaemic stroke (NIHSS of 3 or less) was consistent in the trials of clopidogrel plus aspirin whilst high-risk TIA was defined as an ABCD2 score of 4 or more, or by other high-risk features. In the only study of ticagrelor plus aspirin, people with moderate and minor ischaemic stroke were included (NIHSS of 5 or less) and high risk TIA was defined as either an ABCD2 score of 6 or more (provided motor or speech symptoms were present), or by being accompanied by evidence of symptomatic carotid or intracranial atherosclerotic disease. However, less than 10% of patients were included in the trial due to a TIA. This should be considered when deciding on a dual antiplatelet therapy regimen after minor stroke and TIA.

People with high grade carotid stenosis and planned revascularisation were excluded from POINT, CHANCE and THALES. In addition, people with a clear indication for anticoagulation (such as fibrillation, mechanical heart valve) or recognised increased risk of bleeding were excluded. DAPT should not routinely be used in people with an indication for anticoagulation.

## Loading and maintenance doses

The single loading dose of clopidogrel used in the trials varied from 300 mg to 600 mg. We suggest giving a single loading dose of 300 mg of clopidogrel in patients not already taking the relevant medication, followed by the daily maintenance dose for up to 21 days. The dose of aspirin used in the trials varied from 50 mg to 325 mg and was at the treating physician’s discretion for up to 21 days.

In the THALES study, a 180 mg loading dose of ticagrelor and a single loading dose of 300 or 325 mg of aspirin was used. This was followed by 90 mg twice daily of ticagrelor and 75 to 100 mg of aspirin. We suggest following this dosing regimen.

## Choice of drug regimen

Some people with minor stroke and TIA would have been eligible for the included trials of clopidogrel based DAPT and the trial of ticagrelor based DAPT. Because there are three trials of clopidogrel based DAPT with relevant outcomes and there was no significant increase in intracranial haemorrhage, the level of certainty regarding the available evidence and the strength of the recommendation was rated higher for clopidogrel based DAPT than for ticagrelor based DAPT. However, it should be noted that the sample size of the THALES trial was similar to the combined sample size of the three clopidogrel based trials. In addition, the THALES trial allowed inclusion of people with more severe stroke so the bleeding rate cannot be directly compared across the clopidogrel and ticagrelor trials. DAPT with ticagrelor and aspirin should be considered as an alternative to clopidogrel based DAPT, particularly in people known to be intolerant of clopidogrel or in people who have moderate stroke (NIHSS 4 or 5) and no other contraindication.
